# Demethoxycurcumin Suppresses Human Brain Glioblastoma Multiforme GBM 8401 Cell Xenograft Tumor in Nude Mice *In Vivo*

**DOI:** 10.3390/ijms22115503

**Published:** 2021-05-23

**Authors:** Yi-Ping Huang, Yi-Shih Ma, Chao-Lin Kuo, Ching-Lung Liao, Po-Yuan Chen, Shu-Fen Peng, Fei-Ting Hsu, Kuang-Chi Lai

**Affiliations:** 1 Department of Physiology, School of Medicine, China Medical University, Taichung 406, Taiwan; yphuang@mail.cmu.edu.tw; 2 School of Chinese Medicine for Post-Baccalaureate, I-Shou University, Kaohsiung 840, Taiwan; m2367591@ms25.hinet.net; 3 Department of Chinese Medicine, E-Da Hospital, Kaohsiung 824, Taiwan; 4 Department of Chinese Pharmaceutical Sciences and Chinese Medicine Resources, China Medical University, Taichung 406, Taiwan; clkuo@mail.cmu.edu.tw; 5 College of Chinese Medicine, School of Post-Baccalaureate Chinese Medicine, China Medical University, Taichung 406, Taiwan; qbking@ms29.hinet.net; 6 Department of Biological Science and Technology, China Medical University, Taichung 406, Taiwan; pychen@mail.cmu.edu.tw (P.-Y.C.); t20811@mail.cmuh.org.tw (S.-F.P.); 7 Department of Medical Research, China Medical University Hospital, Taichung 404, Taiwan; 8 Department of Medical Laboratory Science and Biotechnology, College of Medical Technology, Chung Hwa University of Medical Technology, Tainan 717, Taiwan; 9 Department of Surgery, China Medical University Beigang Hospital, Beigang, Yunlin 651, Taiwan

**Keywords:** demethoxycurcumin (DMC), glioblastoma multiforme, xenograft tumor, nude mice, *in* *vivo*

## Abstract

Demethoxycurcumin (DMC), a derivate of curcumin, has been shown to induce apoptotic cell death in human glioblastoma multiforme GBM 8401 cells via cell cycle arrest and induction of cell apoptosis. However, there is no report showing DMC suppresses glioblastoma multiforme cells *in vivo*. In the present study, we investigated the effects of DMC on GBM8401 cells *in vivo*. At first, we established a luciferase-expressing stable clone named GBM 8401/*luc2*. Second, mice were inoculated subcutaneously with GBM 8401/*luc2* cells to generate a xenograft tumor mice model. After inoculation, tumor volume reached 100–120 mm^3^, and all mice were randomly divided into three groups: Group I was treated with 110 µL phosphate-buffered solution (PBS) containing 0.1% dimethyl sulfoxide, Group II with 30 mg/kg of DMC, and Group III with 60 mg/kg of DMC. Mice from each group were given the oral treatment of DMC by gavage for 21 days. The body weight and tumor volume were recorded every 3 days. DMC significantly decreased the tumor volumes, and 60 mg/kg treatment showed a higher decrease in tumor volumes than that of 30 mg/kg, However, DMC did not affect the body weights. The photons emitted from mice tumors were detected with Xenogen IVIS imaging system, DMC at both doses decreased the total photon flux and 60 mg/kg treatment of DMC has low total photon flux than that of 30 mg/kg. The tumor volumes and weights in 60 mg/kg treatment of DMC were lower than that of 30 mg/kg. Immunohistochemical analysis was used to measure protein expression of tumors and results showed that DMC treatment led to lightly staining with anti-Bcl-2 and -XIAP and 60 mg/kg treatment of DMC has lighter staining with anti-Bcl-2 and -XIAP than that of 30 mg/kg. The higher dose (60 mg/kg) of DMC has higher signals of cleaved-caspase-3 than that of the lower dose (30 mg/kg). Furthermore, the hematoxylin and eosin (H&E) staining of liver tissues showed no significant difference between DMC-treated and control-groups. Overall, these observations showed that DMC suppressed tumor properties *in vivo* and DMC may be used against human glioblastoma multiforme in the future.

## 1. Introduction

Gliomas, which originate in the parenchyma of the central nervous system (CNS), are the most common type of adult primary brain tumors. Gliomas are also the most common CNS neoplasms, which are characterized by aggressive growth, high malignant degree, and poor prognosis. There are three subtypes of human gliomas, such as astrocytomas, oligodendrogliomas, and ependymomas. Glioblastoma resides within the category of astrocytoma [1]. The World Health Organization (WHO) divides gliomas into Grade I pilocytic astrocytoma, Grade II diffuse astrocytoma, Grade III anaplastic astrocytoma, and Grade IV glioblastoma [2,3]. Grades III and IV represent the majority of brain tumors [4]. The glioblastoma (GBM) shows a poor prognosis.

Curcuminoids, polyphenol pigment compounds, are the turmeric’s main active ingredients, which are extracted from the *Curcuma longa* rhizome. Curcuminoids contain three major bioactive ingredients, including curcumin (CUR), demethoxycurcumin (DMC), and bisdemethoxycurcumin (BDMC), which are in a ratio of 77:17:3 [5]. CUR has biological activities such as anti-inflammatory, antioxidant, and anti-cancer [6,7,8,9], anti-arthritic and lipid-modifying [10], and analgesic and immune-regulatory [11]. Still, its poor solubility in water and easy degradation *in vitro* and *in vivo* limit its applications [12]. DMC has similar biological properties to CUR, but it is chemically stable [13]. DMC showed the most potent inhibition of excision repair cross-complementation 1 (ERCC1), which plays a significant role in the incision at the 5′ site of damaged DNA from cisplatin treatment when compared to other curcuminoids [14].

Numerous studies have shown that DMC inhibited cell proliferation in many human cancer cells. DMC induced cell apoptosis in human prostate cancer cells, brain malignant glioma GBM 8401 cells, and skin cancer cells [15,16,17]. Besides, it induced DNA damage and apoptosis in human lung cancer NCI-H460 cells and oral cancer SCC-4 cells [18,19,20]. Moreover, DMC was more efficient than TMZ on anti-gliomas and glioma stem cells (GSCs) [21,22,23].

Although DMC has been shown to retard the growth of GBM 8401 cells and induced cell apoptosis *in vitro* [16]. However, there is no available information to show DMC affects GBM 8401 cells *in vivo*. Thus, the current study aims to investigate the effects of DMC on the GBM 8401 cell xenograft mice model *in vivo* and results indicated DMC significantly reduced tumor growth *in vivo*.

## 2. Results

### 2.1. DMC Markedly Inhibited Glioblastoma Tumor Growth

**Figure 1:**
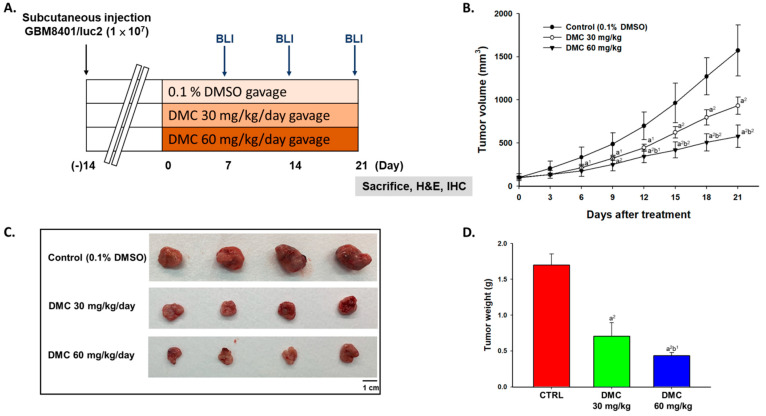
Tumor growth was inhibited by DMC treatment. (**A**) An animal experiment flow chart was displayed. (**B**) Tumor volume was measured by caliper every three days and quantified. (**C**) Tumors were isolated and photographed from each group of mice on day 21. (**D**) Tumor weight was also measured and quantified. (a^1^ *p* < 0.05 and a^2^ *p* < 0.01 vs. control; b^1^ *p* < 0.05 and b^2^ *p* < 0.01 vs. DMC 30 mg/kg).

To examine the anti-tumor effects of DMC, we established human glioblastoma (GBM 8401 cells) bearing animal models. The animal experiment flow chart was displayed in Figure 1A DMC was used to treated glioblastoma mice for 21 days and mouse tumor size was measured by caliper every three days. At the end of treatment, mice were sacrificed and tumors were removed. After treatment of DMC, mouse tumor growth was effectively suppressed and a significant difference was found from day 6 at high dose (60 mg/kg) of DMC treatment (Figure 1B). In Figure 1C,D, tumors were excised, photographed, and weighed on day 21, and the low (30 mg/kg) and high dose (60 mg/kg) of DMC both can reduce the size and weight of tumors.

### 2.2. DMC Markedly Reduced the Signal Intensity of Luc2 from Glioblastoma-Bearing Mice

**Figure 2:**
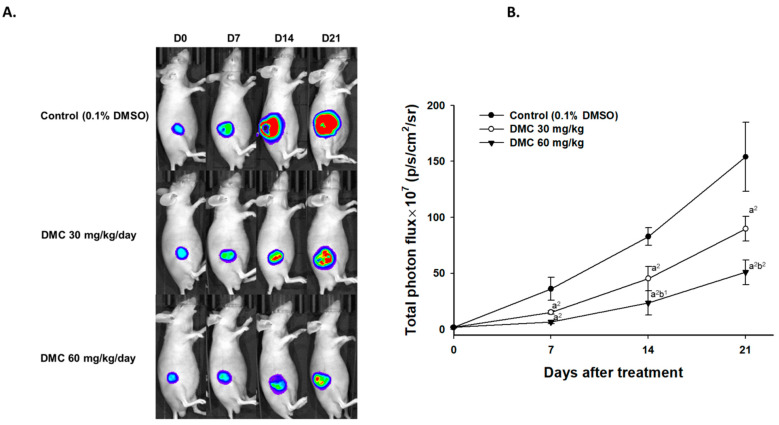
Luc2 signals from living tumor cells were suppressed by DMC treatment. (**A**) The representative BLI results from each group at different time points. (**B**) Quantification results of luc2 signal intensity of tumors. (a^2^ < 0.01 vs. control; b^1^ *p* < 0.05 and b^2^ *p* < 0.01 vs. DMC 30 mg/kg).

Luc2 signal of the tumor, which represents tumor growth, was collected by BLI every week. Figure 2A shows bioluminescent images of mice from each group on days 0, 7, 14, and 21. Luc2 signal intensity from the control group was increased almost 150 times more as compared to day 0 in the control group (Figure 2B). The low dosage (30 mg/kg) of DMC and high dosage (60 mg/kg) both effectively delayed the luc2 intensity growth in glioblastoma tumors. As compared to the low dosage of DMC, the high dosage of DMC showed superior tumor inhibition capacity, showing weak luc2 signals. Furthermore, these results were consistent with the tumor size data.

### 2.3. DMC Suppressed Anti-Apoptosis and Induced Apoptosis Factors in Glioblastoma-Bearing Mice

**Figure 3:**
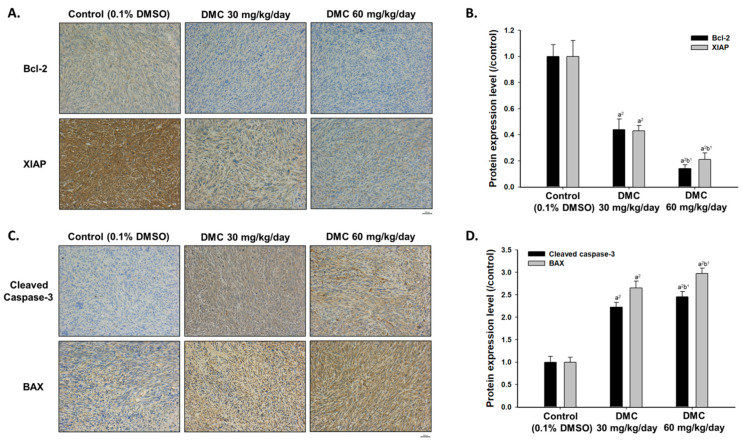
Apoptosis-related proteins were modulated by DMC treatment. (**A**) The IHC staining images of Bcl-2 and XIAP were observed by microscope with 100 times magnification. (**B**) Quantification results of Bcl-2 and XIAP protein levels as compared to control. (**C**) The IHC staining images of cleaved caspase-3 and BAX were observed by microscope with 100 times magnification. (**D**) Quantification results of cleaved caspase-3 and BAX protein levels as compared to control. (a^2^ < 0.01 vs. control; b^1^ *p* < 0.05 vs. DMC 30 mg/kg).

Mice were sacrificed on day 21 and then their tumors were isolated for further validation. Here, we performed IHC staining to investigate the alteration of apoptosis-related markers after DMC treatment. First, we investigated the expression of Bcl-2 and XIAP, both of them were recognized as anti-apoptosis markers, which suppressed the tumor apoptosis effect. After DMC treatment, the protein levels of Bcl-2 and XIAP were decreased around 50–80% as compared to control (Figure 3A,B), indicating DMC decreased anti-apoptosis markers. Besides, we also validated whether apoptosis-related factors were induced at the same time. In Figure 3C,D, the levels of cleaved caspase-3 and BAX were increased two to three times more by DMC as compared to control. The evidence suggested that DMC may suppress tumor growth via regulating apoptosis signaling of glioblastoma.

### 2.4. DMC Treatment Did Not Induce Acute or Delayed Toxicity of Glioblastoma-Bearing Mice

**Figure 4:**
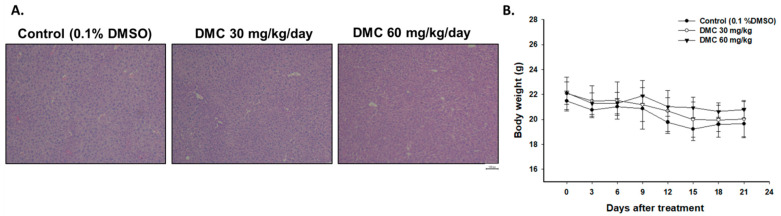
No acute or delayed toxicity was found in DMC-treated glioblastoma-bearing mice. (**A**) Liver pathology photograph from the microscope with 100 times magnification. (**B**) Mouse body weight was measured and recorded every three days.

H&E staining of mice liver was performed in order to identify whether the dosage of DMC used in this study may trigger any toxicity in mice. As shown in Figure 4A, no differences in liver tissues were found in the three groups. Additionally, body weight was also used to monitor general toxicity during the treatment period. In Figure 4B, mouse body weight did not change more than 20% at every time point, indicating no signs of acute or delayed toxicity.

## 3. Discussion

Cancer is still a severe health problem worldwide and it continues to be a leading cause of death in the human population. So far, many plant-derived compounds such as taxol [24], vinblastine [25], and topotecan [26] have been used as anti-cancer drugs for cancer therapy. Around 75% of the clinically used anti-cancer drugs are derived from natural plants, animals, and microorganisms [27], and especially the phytochemicals were used for promising cancer preventative agents and attract research interest [28,29,30]. Numerous studies have shown that DMC induced cell apoptosis in many human cancer cells *in vitro* [15,16,17,18,19,20], including human brain malignant glioma GBM 8401 cells [16]. However, DMC’s anti-tumor activity on the human malignant glioma xenograft mouse model had no available information. Therefore, we used athymic nude mice inoculated with GBM 8401/*luc2* cells for investigating the inhibitory effects of DMC on the growth of GBM 8401 cell xenograft tumor for further clinical use in the future. The whole overall outline of these experiments is shown in Figure 1A.

During the treatment, individual tumor size from each treatment (0, 30, and 60 mg/kg of DMC) was measured by caliper every three days and luc2 signal of the tumor was acquired from BLI every week for treatment of DMC on glioblastoma mice up to 21 days. Results indicated that DMC at both doses significantly reduced tumor volume compared to control and the group at the higher dose of DMC showed higher inhibition of tumor volume than that of the lower (Figure 1B). At the end of treatment, all mice were sacrificed and the tumors were isolated, photographed, and weighted from each mouse of each group and the results are shown in Figure 1C,D. Results indicated that a low dose (30 mg/kg) and a high dose (60 mg/kg) of DMC both could reduce the size and weight of tumors when compared to control (Figure 1C,D). These results indicated that DMC at both doses significantly reduced tumor weights in mice. For the orthotopic glioblastoma xenograft mode, the anti-tumor effects of DMC are not better than temozolomide (the first-line clinical drug for the treatment of brain cancer) [31]. Thus, we will develop nanoparticles loaded with DMC for penetrating the blood-brain barrier.

Twelve mice were inoculated subcutaneously with GBM 8401/*luc2* cells and randomly separated into three groups (control, 30 mg/kg and 60 mg/kg of DMC groups) and followed by Xenogen IVIS imaging system 200 to detect the photons emitted from mice tumor. The Xenogen IVIS imaging system is suitable for examining the effects of test chemicals on inhibiting tumor growth in xenograft animal models [32]. The represented bioluminescent imaging of mice from each group on days 0, 7, 14, and 21 was displayed using the Xenogen IVIS imaging system (Figure 2A). Luc2 signal intensity from the control group increased almost 150 times more than day 0 (Figure 2B). Both doses of DMC effectively delay the Luc2 intensity growth in glioblastoma tumors. The higher dose (60 mg/kg) of DMC has higher inhibition of luc2 signal than the lower dose (30 mg/kg). Furthermore, this result was consistent with tumor size data. These observations indicated DMC inhibited the tumor growth *in vivo*, moreover, DMC significantly reduced tumor volumes and weights (Figure 1B-D) and a higher dose of DMC has a higher inhibition of tumor growth *in vivo*. It was reported that DMC played a major role against heavy metal-induced neurotoxicity and has neuroprotective properties [33]. Herein, results clearly indicated that the administration of DMC (30 and 60 mg/kg) delayed brain tumor development in xenograft animal models. DMC was superior to TMZ in its ability to inhibit cell proliferation and induce apoptosis of GSCs *in vitro* and *in vivo* [34]. Therefore, we may suggest that DMC may have the potential to develop against brain tumors in the future.

In order to further investigate regarding DMC reduced the growth of GBM 8401/*luc2* cell xenograft tumor in nude mice, after treatment, all tumors were collected, stained, and conducted by immunohistochemical analysis. We selected the expressions of anti-apoptotic proteins such as Bcl-2 and X-linked inhibitor of apoptosis (XIAP) and pro-apoptotic proteins such as caspase-3 and Bax for evaluating the DMC’s apoptosis effects. The procaspase levels decreased via the formation of cleaved-caspases during the activation of caspases [35] and apoptosis is regulated by BAX and Bcl-2 (apoptosis-related protein), known as Bcl-2 family members [36]. Results indicated that DMC significantly inhibited the expressions of Bcl-2 and XIAP (Figure 3A,B). Bcl-2 is well documented that is an anti-apoptotic protein and its level decreased will lead to apoptosis. XIAP protein mediates chemotherapy resistance and apoptosis resistance [37] and it is a potent inhibitor of cell death that involved the inhibition of specific caspases [27]. Herein, our findings also showed that XIAP might be one of the mechanisms for reduced tumor volumes and weight in the GBM 8401 cell xenograft tumor mice model. 

Herein, we showed the effects of oral administration of DMC (0, 30, and 60 mg/kg) on the tumor growth of GBM 8401/*luc2* cell xenograft animals and results showed that DMC did not significantly affect the body weights (Figure 4A,B). Furthermore, we examined the liver samples with or without DMC treatment and results indicated no cytotoxicity in liver tissues on all tested animals. Based on these observations, DMC is a suitable and potential compound for further investigation related to anti-glioma *in vivo*.

In summary, overall results showed reduced growth of the GBM 8401 cell xenograft tumor, including the reduction of the tumor volumes and weights by DMC through the induction of apoptosis, based on markedly decreased Bcl-2 and XIAP but significantly increased the cleaved caspase-3 and BAX in tumor tissues from the immunohistochemistry of tumor sections in the DMC treatment groups. Our findings could contribute to a better understanding of human glioblastoma’s molecular mechanisms after exposure to DMC. Thus, it may provide additional targets for developing the new target therapies associated with GBM patient outcomes in the future.

## 4. Materials and Methods

### 4.1. Chemicals and Reagents

Demethoxycurcumin (DMC) was bought from ChemFaces (Wuhan, China) and prepared as 150 mg/mL stock by dimethyl sulfoxide (DMSO) (Sigma Chemical Co., St. Louis, MO, USA). Hygromycin B was obtained from Santa Cruz Biotechnology, Inc. (Dallas, TX, USA).

### 4.2. Cell Culture of Human Glioblastoma GBM8401 Cells

The human glioblastoma cell line (GBM 8401 cells, successfully established from Chinese female with brain glioblastoma multiforme) [38] was obtained from the Food Industry Research and Development Institute (Hsinchu, Taiwan). GBM 8401 cells were maintained in RPMI-1640 (Life Technologies, Carlsbad, CA, USA) containing 10% heat-inactivated fetal bovine serum (FBS) (Hyclone Laboratories, Logan, UT, USA), 2 mM L-glutamine, and antibiotics (100 U/mL penicillin and 100 µg/mL streptomycin) in a 10-cm culture dish in a humidified atmosphere of 5% CO_2_ incubator at 37 °C [39].

### 4.3. Cell Transfection and Stable Clone Selection

Before transfection, confluency of at least 80% of GBM 8401 cells was reached before the transfection procedure. JetPEI^TM^ transfection reagents and pGL4.50 luciferase reporter (pGL4.50 [*luc2*/CMV]) vector were obtained from Polyplus Transfection (Illkirch, Bas-Rhin, France) and Promega (Madison, WI, USA), respectively. According to the manufacture protocol, the detailed transfection and stable clone selection methods were described in our previous studies [40,41]. After transfection, hygromycin B (200 µg/mL) was used to screen and maintain luc2 expression in GBM 8401 cells. GBM 8401 cells with luc2 signals were selected by IVIS 200 Imaging System (Xenogen, Alameda, CA, USA) and identified as GBM 8401/*luc2* cells.

### 4.4. Xenograft GBM 8401 Bearing Animal Model

Twelve male athymic CAnN.Cg-Foxn1nu/CrlNarl (NUDE), 6–8 weeks old, were purchased from the National Laboratory Animal Center (Taipei, Taiwan). The animals were maintained in standard cages in a filtered airflow at 25 °C. China Medical University had approved the study (CMU 2019-204). Animals were allowed to acclimate for 7 days, then GBM 8401/*luc2* cells (1 × 10^7^ cells/mouse) were subcutaneously injected into each mouse’s right flank to form a glioblastoma animal model [40,41].

### 4.5. Treatment and Physical Tumor Growth Validation

The tumor size of each animal was measured every three days using calipers. After the tumor grew for 14 days and its size reached an average of 100 mm^3^, twelve mice were randomly separated into three groups: Control (0.1% DMSO), 30 or 60 mg/kg of DMC groups. The animal study was schematized in Figure 1. Treatment drugs were diluted in 100 µL PBS containing 0.1% DMSO and administered by gavage daily. Tumor volume was measured by digital caliper and calculated by the equation: V = L × W^2^ × 0.523 (where V is the volume, L is the length, and W is the width) [40,41]. Tumors from each mouse were removed, photographed, and weighed after 21 days’ treatment.

### 4.6. In Vivo Bioluminescent Imaging (BLI)

Mice from each group were intraperitoneally injected with 150 mg/kg D luciferin (Promega, Madison, WI, USA), 15 min before being anesthetized using 1–3% isoflurane for BLI scanning. Image acquisition was performed by IVIS 200 Imaging System and luc2 signal intensity was quantified by Living Image software (Version 2.20, Xenogen, Alameda, CA, USA) [41].

### 4.7. Liver Pathology and Tumor Immunohistochemistry Staining

The liver and tumor tissue isolated from mice were fixed with 10% neutral buffered formalin and embedded by paraffin. The liver tissue section stained with hematoxylin and eosin (H&E) was used as an anatomical pathology diagnosis to compare untreated and DMC-treated mice [42]. Tumor immunohistochemistry staining of Bcl-2, XIAP, cleaved caspase-3, and BAX was performed as previously described [41].In brief, tumor sections from the individual group of mice were incubated with primary monoclonal anti-Bcl-2 (1:300 dilution; Cell Signaling, MA, USA), anti-XIAP (1:300 dilution; Elabscience Biotechnology Inc., Houston, TX, USA), anti-cleaved caspase-3 (1:300 dilution; Cell signaling, Danvers, MA, USA), and anti-BAX antibodies (1:300 dilution; Rosemont, IL, USA) at 4 °C overnight. Then, followed with secondary antibodies staining for 1 hr and wash twice by rinse buffer before Horseradish Peroxidase Streptavidin (HRP Streptavidin) inoculation. Finally, slides were dehydrated, stabilized with mounting medium, and scanned by Nikon ECLIPSE Ti-U microscope (×100 magnification, Nikon Instruments Inc., Melville, NY, USA). A total of five view images was quantified by Image J and used to represent the protein level alteration (version 1.50, National Institutes of Health, Bethesda, MD, USA) [43]. Image was quantified by Image J IHC tool box developed. The procedure of analysis was followed by the protocol described on the website (https://imagej.nih.gov/ij/plugins/ihc-toolbox/index.html, since 2014, accessed on 1 January 2021) [44].

### 4.8. Statistical Analysis

Data are all expressed as mean ± standard error. Comparison between two groups was performed using one-way ANOVA by GraphPad prism 7 software (San Diego, CA, USA). The *p* value of less than 0.05 was considered to indicate statistical significance.

## Data Availability

The data generated and analyzed will be made available from the corresponding author on reasonable request.

## Abbreviations

Bcl-2, B-cell lymphoma 2; BDMC, bisdemethoxycurcumin; BLI, bioluminescent imaging; CNS, central nervous system; CUR, curcumin; DMC, demethoxycurcumin; DMSO, dimethyl sulfoxide; ERCC1, excision repair cross-complementation 1; FBS, fetal bovine serum; GBM, glioblastoma; GSCs, glioma stem cells; H&E, hematoxylin and eosin; PBS, phosphate-buffered solution; TMZ, temozolomide; XIAP, X-linked inhibitor of apoptosis protein; WHO, World Health Organization.
